# Plantar Tissue Characteristics in People With Diabetes With and Without Peripheral Neuropathy: A Novel Explanatory Model for DPN Risk Assessment

**DOI:** 10.1111/1753-0407.70094

**Published:** 2025-05-06

**Authors:** Yiming Li, Wei Wu, Liyun Xue, Tianyu Zhao, Yucheng Lu, Xiaohui Qiao, Hong Ding

**Affiliations:** ^1^ Department of Ultrasound Huashan Hospital, Fudan University Shanghai China; ^2^ Department of Endocrine Huashan Hospital, Fudan University Shanghai China; ^3^ Department of Radiology Huashan Hospital, Fudan University Shanghai China; ^4^ National Clinical Research Center for Aging and Medicine Huashan Hospital, Fudan University Shanghai China

**Keywords:** diabetic peripheral neuropathy, plantar, sonoelastography, stiffness, thickness

## Abstract

**Objectives:**

Diabetic peripheral neuropathy (DPN) may affect the biomechanical properties and morphology of the plantar tissue. This study aimed to compare plantar stiffness and thickness in individuals with diabetes with and without DPN and develop a novel explanatory model for DPN risk assessment by integrating these measures with clinical parameters.

**Materials & Methods:**

Thirty‐two healthy controls and 84 people with diabetes (41 with DPN and 43 without DPN) were included. Shear wave elastography evaluated plantar thickness and stiffness at the heel, hallux, and first and fifth metatarsal heads (1st MTH, 5th MTH). An integrated thickness or stiffness index was generated at multiple locations by principal component analysis (PCA).

**Results:**

People with DPN showed a significant increase in plantar thickness (heel, 1st MTH) (*p <* 0.001) and stiffness (all tested locations) compared to healthy controls (*p* < 0.05). Moreover, plantar thickness at 1st MTH, plantar stiffness at 5th MTH, and integrated stiffness index generated by PCA were significantly higher in DPN than in the non‐DPN group (*p* < 0.05). A DPN explanatory model was developed using multivariate logistic regression, incorporating the integrated plantar stiffness index, diabetes duration, and gender. The model showed high discriminative ability (AUROC: 97.7%), with an optimal cutoff of 0.56 yielding 92.7% sensitivity and 95.3% specificity.

**Conclusion:**

The integrated plantar stiffness index, combined with gender and diabetes duration, offers a novel approach for DPN, providing a noninvasive tool for DPN risk assessment.

## Graphical abstract

DPN‐induced gait and postural changes affect the biomechanical properties of plantar tissue, which can be assessed noninvasively by SWE.

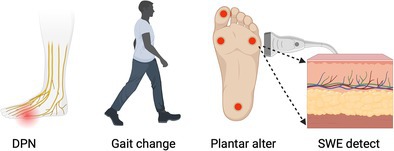

**Summary**

- Shear wave elastography‐based plantar stiffness assessment, integrated with clinical parameters, serves as a screening tool for DPN.

## 1 Introduction

Diabetic foot ulcers, a serious complication in people with diabetes, carry a 25% lifetime risk, with 20% of cases potentially leading to limb amputation, drastically increasing the mortality risk [1, 2, 3]. Diabetic peripheral neuropathy (DPN) is an important risk factor for diabetic foot ulcers, which encompasses sensory, motor, and autonomic neuropathy [1, 4]. Sensory neuropathy leads to loss of protective sensations such as diminished pain, pressure, and temperature sensation, and impaired proprioception. Motor neuropathy causes atrophy of small muscles and deformity of the foot [1, 2, 3, 4].

Currently, no definitive standard exists for DPN, and the diagnosis is based on clinical symptoms and methods of detecting sensory‐motor dysfunction, including the 128 Hz tuning fork to assess vibratory sensation, 10 g Semmes‐Weinstein filament to assess pressure sensation, Achilles tendon reflexes, vibratory sensory threshold testing, and electromyography [1, 3, 5, 6, 7]. Clinical information such as blood glucose, BMI, disease duration, hyperlipidemia, and hypertension was also taken into account [8, 9]. Skin biopsies can detect abnormal nerve density before the onset of sensory nerve dysfunction, meaning that clinical symptoms or detectable nerve deficits may indicate an advanced stage [10]. Although early detection of neuropathy is possible by skin biopsy, its invasive nature prevents its widespread use in clinical practice. Early detection of diabetic neuropathy by noninvasive methods presents a significant challenge.

The soles of the feet contain mechanoreceptors that relay exteroceptive and proprioceptive information regarding body‐environment interaction and body positioning via afferent nerves. This feedback aids in the conscious and reflexive control of balance and movement [11, 12, 13]. Sensory and motor dysfunction caused by neuropathy results in affected control of posture, gait, and balance. Changes in the stimuli to which plantar tissues are exposed during daily activities affect their biomechanical properties and may lead to structural remodeling of plantar tissues [3, 14, 15, 16]. In turn, neuropathy can be assessed by exploring the biomechanical properties and structural changes in plantar tissue [7, 15, 17, 18, 19, 20].

Studies to report plantar tissue stiffening in patients with diabetes or diabetic neuropathy have received more attention [20, 21, 22, 23]. Meanwhile, other reports take the opposite view [15, 24]. Controversy is present regarding the results of changes in plantar tissue thickness in patients with diabetes [21, 25, 26]. Different measurement tools may be a non‐negligible factor in this discrepancy. Ultrasonic palpation systems are non‐visualizable, and the accuracy of the thickness measurement is yet to be investigated. It is also undefined whether the Shore durometer detects full plantar stiffness or superficial skin stiffness [7, 20, 21, 24, 25]. It is essential to use favorable methods to study plantar biomechanics and morphology and explore their link to DPN to comprehend the development of diabetic foot ulcers. Clinical ultrasound, which visualizes detailed structures of the plantar tissue [21, 22], and shear wave elastography, which measures the stiffness of the tissue, offer significant advantages in the noninvasive assessment of plantar features [23, 27, 28]. Given the variable receptor density distribution on different parts of the plantar foot and the varied neuropathy progression rates, changes in plantar tissue properties are not uniform across locations [12, 13]. When studying DPN using plantar tissue properties, it is essential to reference and integrate information from multiple locations [20, 21, 23, 27, 28].

This study aims to compare plantar tissue characteristics in patients with and without DPN and, for the first time, employs principal component analysis (PCA) to integrate data from multiple locations, developing a explanatory model using these characteristics and clinical parameters to identify the presence of DPN.

## 2 Materials and Methods

### 2.1 Participants

The institutional ethical review committee of Huashan Hospital of Fudan University approved the prospective study (HIRB No. 2021‐01030). Informed consent was obtained from the participants. From May 2022 to April 2023, 84 inpatients with diabetes were enrolled in this study, and 32 healthy individuals with no prior history of diabetes were recruited from the community to serve as the control group. Inclusion criteria for participants: age between 18 and 85 years, ability to walk independently, and no other disease‐causing peripheral neuropathy like Guillain–Barre syndrome, disc herniation pressing on nerves, tumors, etc. Exclusion criteria for the healthy control group were as follows: (1) previous diagnosis of diabetes mellitus; and (2) presence of foot ulcers, infections, and amputations. The exclusion criteria for the diabetic group were as follows: (1) a history of foot amputation; (2) the presence of active ulcers on both feet, with tissue edema triggered by acute or subacute inflammation severely affecting the thickness and stiffness of plantar tissue. Patients with diabetes underwent a thorough peripheral nervous system examination by an endocrinologist. The Neuropathy Disability Score (NDS) is utilized to detect peripheral neuropathy. The distribution of NDS scores was shown in Table 1, with a score exceeding 2 signifying neuropathy presence [3]. According to the NDS score, 84 inpatients with diabetes were divided into the DPN group (*n* = 41) and non‐DPN group (*n* = 43).

**TABLE 1:** Demographic characteristics of participants in the HC, non‐DPN, and DPN group.

Variables	HC (*n* = 32)	non‐DPN (*n* = 43)	DPN (*n* = 41)	*p*‐value
Age (y)	54.5 (48–59.75)	59 (51–66)	64 (59–70)	< 0.001^a^, ^b^
Male (%)	17 (53.1)	21 (48.8)	32 (78)	0.015^b^
Height (cm)	165.63 ± 6.40	165.81 ± 8.64	168.73 ± 7.88	0.146
Weight (kg)	68.59 ± 6.88	72.96 ± 14.09	69.10 ± 11.26	0.182
BMI (kg/m^2^)	25.03 ± 2.32	26.39 ± 3.81	24.24 ± 3.58	0.015^b^
Hypertension (%)	/	26 (60.5)	25 (61)	0.962
Smoke (%)	/	12 (27.9)	13 (31.7)	0.703
NDS score (n)				
0–2	/	43	/	
3–5	/	/	18	
6–8	/	/	18	
9–10	/	/	5	
Foot ulcers	/	1	10	0.008

*Note:* Data are represented as mean ± SD or median (interquartile ranges).

Abbreviations: DPN, patient with diabetic peripheral neuropathy; HC, health control; non‐DPN, patient without diabetic peripheral neuropathy.

^a^ Indicates a significant difference between HC and DPN (*p* < 0.001).

^b^ Indicates a significant difference between non‐DPN and DPN (*p* < 0.05).

### 2.2 Thickness and Stiffness Measurement of Plantar Tissue

All images were acquired using the Cannon Aplio i900 ultrasound (Cannon Medical Systems, Japan), fitted with a broadband linear array probe of 5–18 MHz frequency, designed for musculoskeletal applications. To avoid the influence of extension or flexion on plantar tissue properties, the examination was performed with the subject in a lateral position with the knee flexed and the ankle relaxed, and the subject's plantar tissue facing or facing back to the examiner. All measurements were conducted by Dr. Li, a highly experienced sonographer with a decade of expertise in clinical ultrasound and specialized knowledge in advanced musculoskeletal ultrasound, who holds an accreditation in the field. Real‐time imaging was used to observe the plantar structure and determine the measurement locations, which included the heel, hallux, first metatarsal head (1st MTH), and fifth metatarsal head (5th MTH) on the longitudinal imaging plane across four plantar foot locations. These sites, more prone to higher plantar pressure and diabetic foot ulcer development, were where plantar tissue thickness (mm) and stiffness (in terms of shear wave speed; m/s) were measured. The transducer was gently positioned on the sole in a non‐loaded state to prevent tissue compression or deformation resulting from transducer pressure.

**FIGURE 1:**
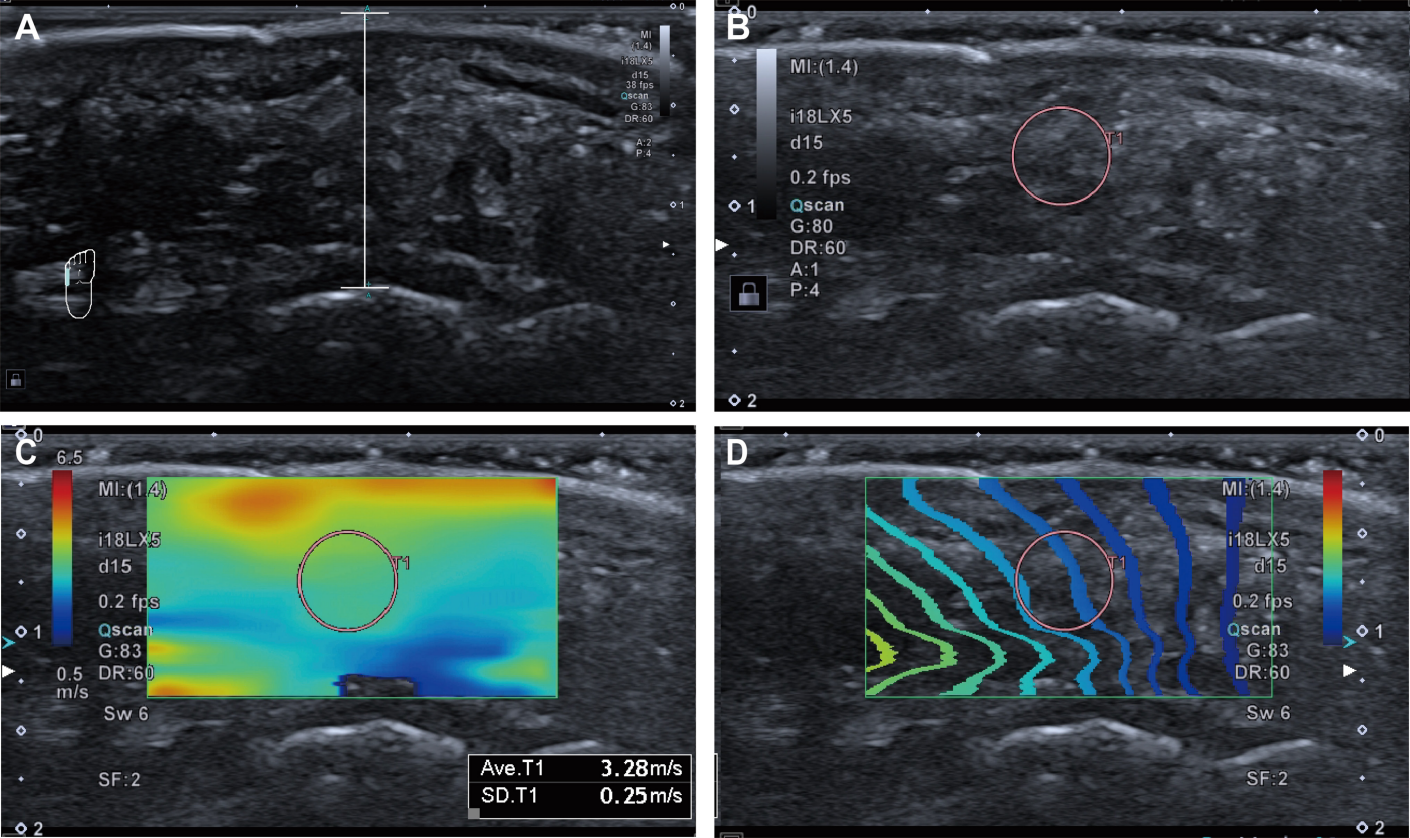
Measurement of thickness and stiffness of the plantar tissue at 5th MTH. (A) Plantar tissue thickness was measured on the gray‐scale image. (B) Original gray‐scale image for elastography (C) The regions of interest (circle) were selected on the elastography mode to measure the plantar stiffness in terms of shear wave speed. (D) Shear wave elastography quality control map.

The thickness measurement referred to the distance between the skin surface and the corresponding bone cortical convexity in the image. Stiffness measurements were conducted in shear wave elastography (SWE) mode, with color elastography overlaid on the corresponding B‐mode images. The elastography sampling frame included the skin and bone cortex at corresponding locations. Circular regions of interest (ROI) with size 5 were traced at the center of the elastography sampling frame, avoiding areas near the skin and bone cortex that are more susceptible to artifacts from shear wave boundary effects or multiple reflection‐induced wave superimposition, as shown in Figure 1. The stiffness of plantar tissue at each location was defined as the mean shear wave speed within the corresponding ROI. In the case of subjects without foot ulcers, data was gathered from both feet and then averaged. However, for those with active ulcers on one foot, considering that inflammation can affect the plantar nature and the characteristics of bilateral involvement of DPN, measurements were taken solely from the unaffected foot.

### 2.3 Statistical Analysis

All statistical analyses were undertaken using SPSS (Version 25, IBM, Armonk, NY, USA). Statistical significance was set at *p* < 0.05. The Shapiro–Wilk test checked all continuous parameters' normal distribution. Data were expressed as mean ± SD if normally distributed, and median (interquartile ranges) if otherwise.

Differences in demographic data, plantar thickness, and stiffness between groups (HC/non‐DPN/DPN) were analyzed using ANOVA or the Kruskal‐Wallis rank sum test. Sex ratios were evaluated with Pearson's Chi‐squared test. Either the Mann–Whitney U‐test or t‐test was used for comparing clinical parameters between the DPN and non‐DPN groups. The Kruskal‐Wallis rank sum test examined variations in thickness or stiffness among the four different locations.

The PCA technique was utilized to downscale and integrate thickness or stiffness measurements at the four plantar locations. PCA involves initially deriving an orthogonal matrix, followed by applying an orthogonal transformation to the data to yield new datasets, as formula(1). The PCA model is based on the following model (2). The proportion of data variance accounted for by the newly derived principal components is articulated in Equation (3). PCA was used to generate integrated plantar thickness and stiffness indexes (ZTH, ZSWE), and the indexes with high eigenvalues were selected to reflect the comprehensive information.

(1) Z=BX

Where $B=\left[\begin{array}{cc} \begin{array}{cc} \beta_{11} & \beta_{12}\\ \beta_{21} & \ddots \end{array} & \begin{array}{cc} \ldots & \beta_{1N} \end{array}\\ \begin{array}{cc} \vdots & \\ \beta_{N1} & \beta_{N2} \end{array} & \begin{array}{cc} \ddots & \\ \cdots & \beta_{\mathrm{NN}} \end{array} \end{array}\right]$

(2) $$Z_{\mathrm{ij}}=\sum_{k=1}^{p}\beta_{\mathrm{ik}}X_{\mathrm{kj}}$$

where *i, j = 1, 2, 3*, …, *p*

(3) $$R^{2}=\frac{\sum_{j=1}^{K}\lambda_{j}}{\sum_{j=1}^{N}\lambda_{j}}$$

where λ present variance.

We used univariate logistic regression models for clinical and ultrasonic parameters to identify independent variables that could aid in DPN risk. Multivariate logistic regression then incorporated parameters with *p*‐values less than 0.1. Variables significant at *p* < 0.05 were included in the model. The model's accuracy in DPN was evaluated using the ROC curve, with the Youden index identifying the best cutoff point.

### 2.4 Results

A comparison of demographics in the different groups is shown in Table 1. The DPN group was significantly older compared to the non‐DPN (*p =* 0.006) and HC groups (*p <* 0.001). Concurrently, patients with DPN tended to include a higher percentage of males compared to those without DPN (*p =* 0.006). The DPN group exhibited a significantly lower BMI compared to the non‐DPN group; however, there were no significant differences in height and weight between these groups(*p* > 0.05).

#### 2.4.1 Thickness and Stiffness of Plantar Tissue

The results regarding the thickness and stiffness of the plantar tissues among the three groups were displayed in Table 2. Plantar tissue thickness at the heel and 1st MTH and stiffness at all test locations were different among the three groups. Increased thickness and stiffness of plantar tissue are more pronounced in patients with DPN compared to healthy controls. Plantar tissue thickness differed between DPN and non‐DPN groups only at the 1st MTH (*p* < 0.05). Interestingly, the trend in plantar tissue stiffness was more pronounced in diabetic neuropathy, where patients with DPN showed higher plantar tissue stiffness than healthy controls at all tested locations (*p* < 0.05). However, there was a significant difference in plantar tissue stiffness between DPN and non‐DPN groups only at the 5th MTH (*p* < 0.05). The median shear wave velocity was 3.9 m/s and 3.41 m/s, respectively.

**TABLE 2:** The comparison of thickness and stiffness at different locations of plantar tissue between HC, non‐DPN, and DPN groups.

Location	Group	Thickness (mm)	*p*‐value statistics	Pairwise comparisons	*p*‐value statistics	shear wave speed (m/s)	*p*‐value statistics	Pairwise comparisons	*p*‐value statistics
Heel	HC	1.54 ± 0.19	< 0.001	HC vs. non‐DPN	0.001	2.63 (2.39–2.84)	< 0.001	HC vs. non‐DPN	< 0.001
	non‐DPN	1.72 ± 0.23		non‐DPN vs. DPN	0.403	3.37 (2.83–4.03)		non‐DPN vs. DPN	0.783
	DPN	1.76 ± 0.22		HC vs. DPN	< 0.001	3.49 (3.08–4.30)		HC vs. DPN	< 0.001
Hallux	HC	1.14 ± 0.12	0.6	HC vs. non‐DPN	0.891	2.50 (2.31–2.72)	0.012	HC vs. non‐DPN	0.981
	non‐DPN	1.14 ± 0.14		non‐DPN vs. DPN	0.342	2.54 (2.32–2.87)		non‐DPN vs. DPN	0.121
	DPN	1.17 ± 0.16		HC vs. DPN	0.456	2.80 (2.50–3.01)		HC vs. DPN	0.012
1st MTH	HC	0.99 ± 0.07	0.001	HC vs. non‐DPN	0.085	2.97 (2.52–3.34)	0.088	HC vs. non‐DPN	0.094
	non‐DPN	1.05 ± 0.16		non‐DPN vs. DPN	0.017	3.19 (2.74–3.78)		non‐DPN vs. DPN	0.546
	DPN	1.13 ± 0.19		HC vs. DPN	< 0.001	3.34 (2.75–4.31)		HC vs. DPN	0.036
5th MTH	HC	1.17 ± 0.16	0.254	HC vs. non‐DPN	0.197	2.79 (2.59–3.35)	< 0.001	HC vs. non‐DPN	0.001
	non‐DPN	1.22 ± 0.16		non‐DPN vs. DPN	0.131	3.41 (2.90–3.93)		non‐DPN vs. DPN	0.036
	DPN	1.16 ± 0.18		HC vs. DPN	0.904	3.9 (3.38–5.12)		HC vs. DPN	< 0.001

*Note:* Sample size of the groups: HC *n* = 32, non‐DPN *n* = 43 and DPN *n* = 41. Data are represented as mean ± SD or median (interquartile ranges).

Abbreviations: 1st MTH, First metatarsal head; 5th MTH, Fifth metatarsal head; DPN, people with diabetic peripheral neuropathy; HC, health control; non‐DPN, people without diabetic peripheral neuropathy.

#### 2.4.2 Differences in Plantar Tissue Thickness and Stiffness at Multiple Locations

**FIGURE 2:**
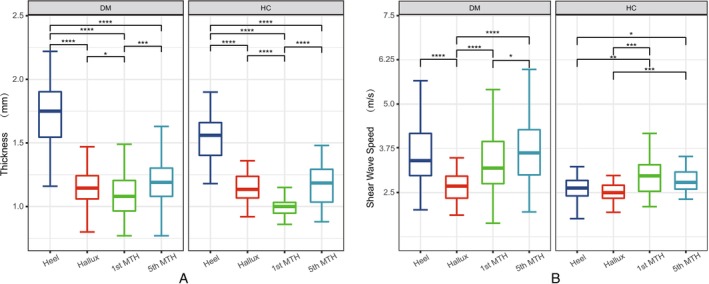
Thickness and stiffness of plantar tissue at different locations. (A) Thickness of plantar tissue at different locations. (B) Stiffness of plantar tissue at different locations; MTH, Metatarsal head; HC, Healthy control; DM, Diabetes mellitus.

There were significant differences in plantar thickness and stiffness in different locations, as shown in Figure 2. The thickness and stiffness of the plantar tissue were more heterogeneous in people with diabetes compared to healthy individuals.

#### 2.4.3 Integrated Stiffness and Thickness Indexes of Plantar Tissue by PCA

In order to unite the thickness and stiffness properties of plantar tissues in different locations so as to reduce the dimensionality of the original data, and at the same time to avoid the loss of useful information, PCA was used to generate integrated thickness and stiffness indexes that can reflect the thickness and stiffness attributes of plantar tissues in multiple locations according to the different weights, so as to comprehensively reflect the characteristics of plantar tissues. Retain the two integrated thickness indexes (Z1TH, Z2TH) and the integrated stiffness indexes (Z1SWE, Z2SWE) of plantar tissue with larger eigenvalues, respectively. Z1TH and Z2TH accounted for 58.16% and 18.68% of the variance, respectively, explaining 76.84% of the total variance in the integrated plantar tissue thickness characteristics. Z1SWE and Z2SWE accounted for 49.07% and 22.41% of the variance, respectively, explaining 71.48% of the total variance in the integrated plantar tissue stiffness characteristics. U‐tests for differences in Z1TH, Z2TH, Z1SWE, and Z2SWE between the patients with and without DPN showed a significant difference only for Z1SWE (*p* < 0.05).

#### 2.4.4 Logistic Regression Analysis for Risk Factors Associated With DPN

**FIGURE 3:**
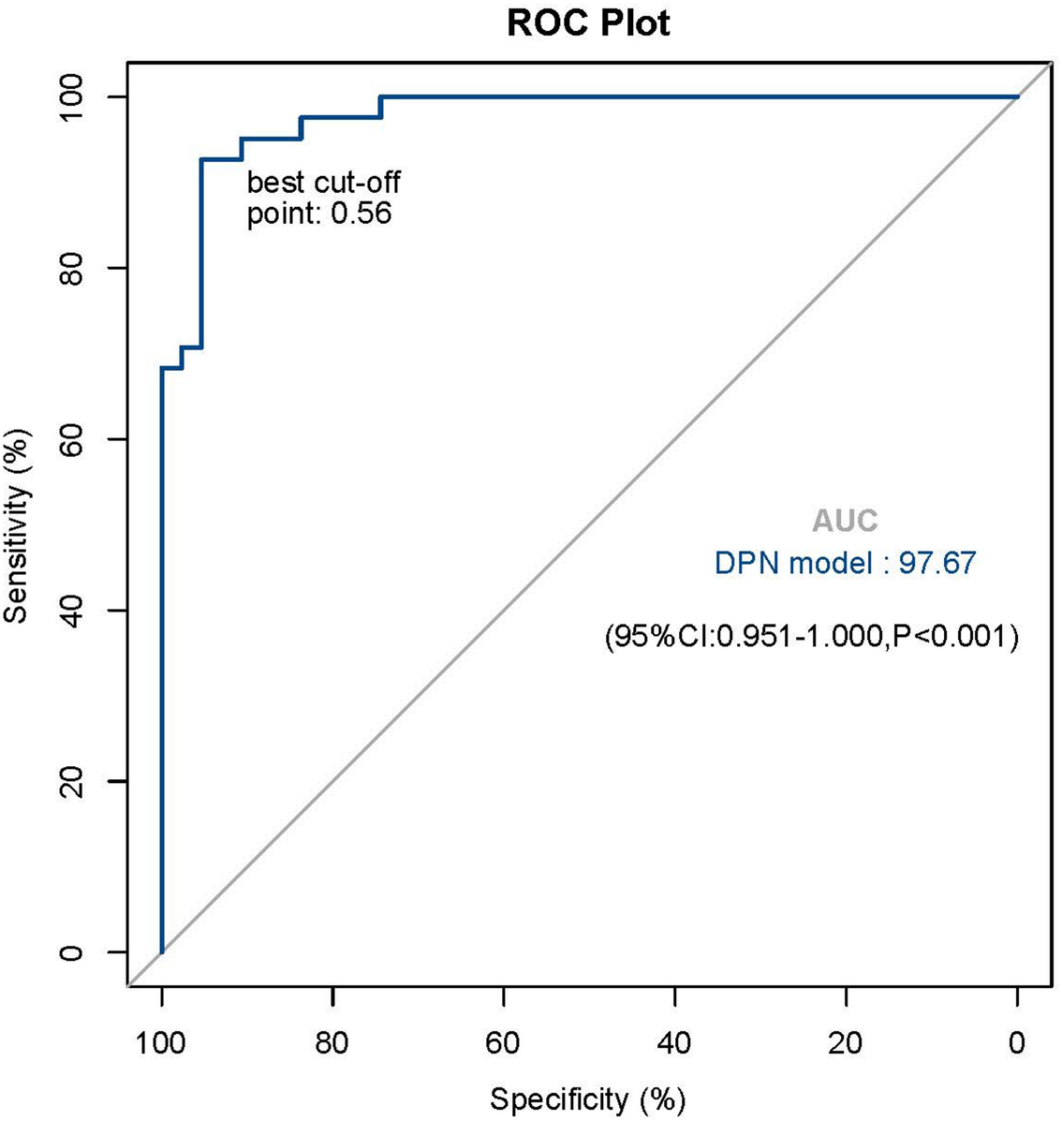
ROC curve of a comprehensive diagnostic model for DPN.

Results of the univariate and multivariate logistic regression analysis for DPN are shown in Table 3. The univariate analysis showed that having an older age, a lower BMI, a longer duration of DM, increased cholesterol, male gender, a lower integrated thickness index (Z2TH), and a higher integrated stiffness index (Z1SWE) significantly increased the odds for DPN. Furthermore, in the multivariate analysis, a higher integrated stiffness index (odds ratio [OR] 2.79 [95% CI 1.27–6.12]), longer duration of diabetes (1.68 [1.31–1.91]), and gender(women contrast men) (0.09 [0.01–0.75]) remained independently significantly related to DPN. Based on these results, the estimated logistic regression equation was defined as: logit(*p*) = −6.451 to 2.437 (men) + 0.526 (duration of diabetes) + 1.027 (Z1SWE). The ROC curve was plotted based on the explanatory probability of the multivariate logistic regression model, and the area under the ROC curve was 97.7%; in addition, the optimal probability cutoff value of 0.56 yielded a 92.7% sensitivity (correctly classifying the group with DPN) and 95.3% specificity (correctly classifying the group without DPN) for this model (Figure 3).

**TABLE 3:** Univariate and multivariate logistic regression model of DPN.

Variables	non‐DPN (*n* = 43)	DPN (*n* = 41)	*p*‐value univariate analyses	*p*‐ value multivariate analyses	OR value (95 CI%)
Age (y)	59 (51–66)	64 (59–70)	0.004*		
BMI (kg/m^2^)	26.39 ± 3.81	24.24 ± 3.58	0.014*		
Duration (y)	2 (0.58–6)	15 (12–22)	< 0.001*	< 0.001	1.676 (1.313–1.909)
HbA1C (%)	7.6 (6.5–10)	8.2 (7.3–10.1)	0.138		
FBG (mg/dL)	7.7 (6.1–9.1)	7.7 (6.1–9.1)	0.470		
PBG (mg/dL)	11.6 5 ± 4.48	11.18 ± 3.94	0.610		
Cholesterol (mg/dL)	4.47 ± 1.07	3.85 ± 1.41	0.032*		
Triglyceride (mg/dL)	1.73 (1.02–2.68)	1.28 (0.92–1.88)	0.317		
Male (%)	21 (48.8)	32 (78)	0.007*	0.026	0.087 (0.01–0.747)
Z1TH	−0.0883 (−1.0102 ~ 0.9708)	0.2793 (−0.6696 ~ 0.7319)	0.517		
Z2TH	−0.1165 (−0.7746 ~ 0.4962)	−0.1646 (−0.4216 ~ 0.4372)	0.079*		
Z1SWE	−0.4599 (−1.1839 ~ 0.3235)	0.0714 (−0.6497 ~ 1.3959)	0.020*	0.010	2.792 (1.274–6.119)
Z2SWE	−0.0470 (−0.5323 ~ 0.3885)	−0.0414 (−0.6311 ~ 0.8165)	0.913		

Abbreviations: DPN, patient with diabetic peripheral neuropathy; non‐DPN, patient without diabetic peripheral neuropathy; Z1TH/Z2TH, integrated thickness index generated by principal component analysis (PCA); Z1WSE/Z2WSE, integrated stiffness index generated by PCA.

*p < 0.1.

### 2.5 Discussion

Our study investigated differences in plantar tissue thickness and stiffness among individuals with diabetes (with and without DPN) and healthy controls, highlighting the potential of these measures for DPN. We constructed integrated thickness and stiffness indexes that comprehensively capture the characteristics of plantar tissues by using PCA. Furthermore, the model developed in this study, which combines the plantar stiffness index with gender and diabetes duration, provides a robust and noninvasive tool for identifying DPN in individuals with diabetes.

Several studies report conflicting findings on plantar tissue thickness changes in people with diabetes. Our study found an increased overall plantar tissue thickness in people with diabetes, more so in those with neuropathic complications. Notably, significant differences in plantar tissue thickness at the 1st MTH and heel were observed between some groups. This corresponds with Chao's findings, revealing thicker plantar tissue in people with diabetes and increased thickening at more locations, including the 3rd and 5th MTH and the hallux [25]. It is worth mentioning that they used an ultrasonic palpation system to measure the thickness of the plantar tissue, a method that does not allow for visualization and therefore may not be as precise as ultrasound when choosing where to measure. This approach was also used in a study by Sun [21], which showed no significant difference in plantar thickness between healthy individuals and those with diabetic neuropathy. Plantar soft tissues are rich in collagen. Therefore, collagen components are susceptible to non‐enzymatic glycation. Increased tissue thickness in people with diabetes is thought to be associated with a hyperglycemic state, which promotes non‐enzymatic glycation of structural proteins in collagen, elastin, and keratin, which promotes intermolecular cross‐linking of soft tissues, which in turn leads to tissue thickening [29, 30, 31, 32]. Meanwhile, other studies have found the exact opposite results. The thickness of the heel and the first and second metatarsal heads was greater in healthy controls than in diabetic patients, who in turn had greater foot thickness than those with ulcers. Based on this result, the study hypothesized that the loss of tissue mass in the feet of people with diabetes contributed to their susceptibility to foot ulcers [33]. This discrepancy could potentially be attributed to the fact that plantar tissue thickness is impacted by additional factors, such as BMI and age [29, 34].

Stiffness, indicating the elasticity of a material when a compressive force is applied, is reflective of the biomechanical properties of soft tissues. Alterations in the stiffness of plantar tissues can interfere with their natural function as shock reducers and absorbers [20, 21, 35]. Our research found that the DPN group exhibited significantly stiffer plantar tissues at all measured locations compared to the HC group. These findings align with previous studies assessing plantar stiffness using ultrasound palpation systems and Shore durometers [7, 20, 21, 25, 34]. Sun's [21] results, closely mirroring ours. Meanwhile, Chao's [25] research highlighted that plantar stiffness was more pronounced in patients with advanced progression of diabetic ulcers. Duan's [20] study revealed that the tissue stiffness in the hallux, forefoot, midfoot, and rearfoot regions was notably higher in DPN patients compared to non‐DPN patients when the patient's activity load was elevated (*p* < 0.05). The findings from all the aforementioned studies collectively indicate that the severity of plantar stiffness intensifies with the progression of diabetes and peripheral neuropathy. In contrast, Naemi et al. [24] utilized real‐time strain elastography, revealing a lower relative heel stiffness in ulcerated patients compared to nonulcerated. They measured heel stiffness at the non‐ulcer site in patients with active ulcers, and it is worth discussing that the tissue edema from acute inflammation during ulcer presentation could distort the true heel stiffness. Also, strain elastography's repeatability and stability are inferior to SWE.

Numerous studies highlighted the link between plantar stiffness changes and the glycosylation response in those at risk for diabetic foot. Glycosylation, a glucose‐mediated intermolecular cross‐linking process, contributes significantly to tissue aging [32]. These crosslinks typically result in tissue dysfunction, reducing critical flexibility and permeability, and decreasing metabolism [30]. It is the alteration of tissue properties by glycation (e.g., causing the tissue to stiffen) that triggers many devastating late complications [10, 23, 30, 31].

Another category of research emphasizes that changes in plantar stiffness are closely related to plantar loading [20, 35, 36]. If plantar stress exceeds the tissue's carrying capacity, injury can result. From an engineering perspective, tissue damage alters its mechanical properties, which means that changes in tissue biomechanics as a result of damage can be used to detect injury. An in vivo model devised by Chatzistergos found that repeated loading to the pain threshold resulted in statistically significant persistent stiffness in the overloaded area. Whereas the pain threshold is raised when neuropathy occurs, prolonged overloading of the plantar foot is destined to stiffen the tissue [35]. In healthy populations, runners with greater plantar loading have softer heels compared to cyclists [36], but studies in people with diabetes have shown the opposite [20]. This disparity suggests that plantar tissue characteristics may be altered in people with diabetes, who have stiffer plantar tissue, which reduces the ability to distribute pressure over a wider area to reduce high stress, leading to reduced adaptive capacity.

Similar to other studies, patients with DPN had significantly higher plantar tissue stiffness at multiple sites compared to healthy controls [21, 25]. However, the locations of significant differences in plantar stiffness between people with or without neuropathy in diabetes were inconsistent, with only the 5th MTH showing higher stiffness in the DPN group in our study. Differences in plantar nerve distribution may explain this phenomenon to some degree [11, 12, 13]. Strzalkowski summarized the estimates of plantar innervation density with a synthesis of the relevant literature, concluding that the highest density of plantar innervation was in the toes (23.3 units/cm^2^), followed by the lateral arch (15.4 units/cm^2^) and the lateral metatarsals (11.2 units/cm^2^) [12]. In this study, the 5th metatarsal head belongs to one of the regions with the highest density of nerve distribution, and its changes may be most pronounced when neuropathy occurs, as we found, leading to the plantar tissues stiffening first. Some studies have been designed to sum the plantar stiffness values from multiple locations of the plantar to consider the plantar as a whole for research purposes [20]. Based on the theory that the differences in nerve distribution [12, 13] and the unbalanced degree of denervation produced during the development of diabetic neuropathy [37], it can be inferred that the changes in the biomechanical properties of the plantar foot are also uneven. At the same time, our findings indicated significant differences in thickness and stiffness measurements across four different plantar tissue sites. Variability is more pronounced in people with diabetes compared to healthy individuals. Therefore, when studying the biomechanical properties of the plantar tissues, it may be more meaningful to consider the plantar tissues as a whole and take into account the weighting of the different locations.

Our study is the first to employ PCA to integrate thickness and stiffness data from multiple plantar locations by projecting the data onto principal feature vectors, thereby reducing the dimensionality of the original dataset while preserving useful information and providing a more comprehensive assessment of tissue characteristics. The PCA technique combines subsets of highly correlated but distinct variables to form factors that summarize the information contained in a continuous multivariate data set [38, 39]. The integrated stiffness index Z1SWE, which explained 49.07% of the total variance of the stiffness variables of plantar tissue, differed significantly between the DPN and DM groups (*p* = 0.02). Z1SWE integrated the stiffness of four tested locations, which, to some extent, reflects the stiffness parameters of the entire foot and is more valuable than a single site.

Clinical parameters are of undeniable importance in screening populations at high risk for diabetes complications [4, 40, 41]. In our univariate logistic regression model, age, gender, BMI, duration of diabetes, and cholesterol level were significant risk factors for DPN. Research on diabetic foot ulcers summarized that age was the first‐degree risk factor, and male and duration were third‐degree risk factors [3, 4, 41]. A population‐based cross‐sectional study in China examining the prevalence and risk factors of DPN found that patients with DPN were significantly older compared to those without DPN [median age (Q1, Q3) was 63 (55, 71) years versus 59 (50, 67) years, *p* < 0.001]. They also had a longer duration of diabetes [median duration (Q1, Q3) was 8 (5, 14) years versus 6 (3, 11) years, *p* < 0.001], and a higher proportion of males was observed (38.4% vs. 19.2%, *p* < 0.002) [42]. The age‐standardized male‐to‐female ratio of years lived with disability due to neuropathy, amputation, and foot ulcers increased progressively, rising from 0.96 to 1.93, further indicating a higher risk among men compared to women [43]. Oguejiofor et al. showed that 100% of diabetic patients with a disease duration of more than 15 years had peripheral neuropathy, regardless of whether they had symptoms of neuropathy [40]. Skin biopsies directly showed that intraepidermal nerve fiber density was negatively correlated with the duration of diabetes [10]. Age, hyperglycemia status, and duration of diabetes could accelerate tissue aging, probably all based on the process of glycation, which is a major cause of tissue dysfunction [29, 30].

The integration of plantar stiffness index with gender and diabetes duration offers a novel and comprehensive approach for explaining DPN. The model shows that a one‐unit increase in Z1SWE in plantar tissue raises the chances of developing DPN by 179% (i.e., EP(b) = 2.792), and a one‐year increase in diabetes duration ups the risk by 68% (i.e., EP(b) = 1.676). Men with diabetes are 11.5 times more susceptible to DPN compared to women (i.e., EP(b) = 0.087). This noninvasive method could serve as a valuable tool for DPN risk assessment in clinical practice, particularly in resource‐limited settings where advanced diagnostic tools may not be readily available. By identifying individuals at high risk of DPN, this model could facilitate timely interventions, potentially reducing the incidence of DPN‐related complications such as foot ulcers and amputations.

This study has several limitations. First, although it is the first to integrate stiffness information from four plantar sites to and explain DPN, the principal components accounted for only 49.07% of the total stiffness information, likely due to the limited number of sites measured. Second, this study has a cross‐sectional design, limiting its findings to explaining the presence of DPN and its associated factors at the time of data collection, without establishing causality or predictive relationships. Additionally, the small sample size may affect the generalizability of the results. Future longitudinal and larger multi‐center studies are needed to validate the model, establish temporal relationships, and further explore the link between plantar biomechanical properties and DPN.

In conclusion, this study revealed that individuals with diabetes, particularly those with DPN, exhibit thicker and stiffer plantar tissues at specific locations compared to healthy controls. The integrated stiffness index, derived from multiple plantar locations through PCA, effectively distinguished between individuals with and without DPN. By incorporating clinical parameters such as diabetes duration and gender, we identified that longer diabetes duration, male sex, and a higher integrated stiffness index are associated with an increased likelihood of DPN. These findings suggest that plantar tissue characteristics, combined with clinical data, provide a comprehensive approach for describing and explaining DPN. From a broader perspective, the integration of readily obtainable clinical parameters and plantar stiffness measurements offers a potential screening tool for DPN, which could enhance the precision of prevention and intervention efforts, particularly for high‐risk populations.

## Disclosure

The authors have nothing to report.

## Ethics Statement

Institutional Review Board approval was obtained at Huashan Hospital of Fudan University (HIRB No. 2021‐01030).

## Consent

Written informed consent was obtained from all subjects (patients) in this study.

## Conflicts of Interest

The authors declare no conflicts of interest.

## Data Availability

All relevant data are within the paper.
